# Functional Microbial Features Driving Community Assembly During Seed Germination and Emergence

**DOI:** 10.3389/fpls.2018.00902

**Published:** 2018-06-29

**Authors:** Gloria Torres-Cortés, Sophie Bonneau, Olivier Bouchez, Clémence Genthon, Martial Briand, Marie-Agnès Jacques, Matthieu Barret

**Affiliations:** ^1^IRHS, INRA, Agrocampus Ouest, Université d’Angers, Beaucouzé, France; ^2^INRA, US 1426, Castanet-Tolosan, France

**Keywords:** seed microbiome, copiotrophy, seed germination, metagenomics, plant microbial communities

## Abstract

Microbial interactions occurring on and around seeds are especially important for plant fitness since seed-borne microorganisms are the initial source of inoculum for the plant microbiota. In this study, we analyze structural and functional changes occurring within the plant microbiota at these early stages of the plant cycle, namely germination and emergence. To this purpose, we performed shotgun DNA sequencing of microbial assemblages associated to seeds, germinating seeds and seedlings of two plant species: bean and radish. We observed an enrichment of *Enterobacteriales* and *Pseudomonadales* during emergence and a set of functional traits linked to copiotrophy that could be responsible for this selection as a result of an increase of nutrient availability after germination. Representative bacterial isolates of taxa that are selected in seedlings showed indeed faster bacterial growth rate in comparison to seed-associated bacteria isolates. Finally, binning of metagenomics contigs results in the reconstruction of population genomes of the major bacterial taxa associated to the samples. Together, our results demonstrate that, although seed microbiota varied across plant species, nutrient availability during germination elicits changes of the composition of microbial communities by potentially selecting microbial groups with functional traits linked to copiotrophy. The data presented here represents the first attempts to empirically assess changes in the microbial community during plant emergence and moves us toward a more holistic understanding of the plant microbiome.

## Introduction

Plants carry diverse microbial assemblages that can influence different plant features like disease resistance (Santhanam et al., 2015; Busby et al., 2016; Ritpitakphong et al., 2016), flowering time (Panke-Buisse et al., 2014; Dombrowski et al., 2017) or biomass accumulation (Sugiyama et al., 2013). The promotion of these plant traits by manipulation of microbial assemblages requires a fundamental knowledge on the processes that drive the assembly of the plant microbiota. To date, this fundamental knowledge has been mostly gained in two plant habitats: the phyllosphere and the rhizosphere (Müller et al., 2016). The choice of these two compartments stems in part from the postulate that members of the plant microbiota are horizontally acquired from the surrounding environments. For instance, aerosols and rainfall have been suggested as major sources of leaf inoculum (Lymperopoulou et al., 2016; Vacher et al., 2016). Furthermore, a two-step selection process has been proposed for explaining assembly of the root microbiota, where rhizodeposits are responsible for recruitment of a pool of soil-derived microorganisms within the rhizosphere and host-specific factors subsequently selected some members of this pool in the rhizoplane and endosphere (Bulgarelli et al., 2013).

In contrast to horizontal transmission, the relative importance of vertical transmission, which is the acquisition of microbial entity from the parent, in the assembly of the plant microbiota has been relatively unexplored (Shade et al., 2017). However, vertical transmission of fungi and bacteria has been frequently reported in various plant species (Hardoim et al., 2015; Truyens et al., 2015). In that respect, seed-associated microbial assemblages are ecologically interesting because they both represent an endpoint and a starting point for community assembly of the plant microbiota (Shade et al., 2017). Seed-associated microbial assemblages are composed of early microbial colonizers that are acquired from the mother plant either through the vascular system or the stigma and late colonizers incorporated *via* the contact of the seed with microorganisms present on fruits or threshing residues (Maude, 1996). According to diversity surveys performed on seeds of various plant species, these assemblages are composed of bacterial and fungal taxa frequently observed in other plant habitats such as *Proteobacteria*, *Actinobacteria*, *Firmicutes*, or *Dothideomycetes* (Lopez-Velasco et al., 2013; Links et al., 2014; Barret et al., 2015; Adam et al., 2016; Klaedtke et al., 2016; Rezki et al., 2016; Rybakova et al., 2017).

In addition to their potential impact on the assembly of the plant microbiota, seed-associated microbial assemblages can have direct consequences on seed and seedling growth and health. Indeed, the composition of the seed microbiota can contribute to seed preservation (Chee-Sanford et al., 2006), release of seed dormancy (Goggin et al., 2015) and increase or decrease of germination rate (Nelson, 2017). Moreover, seed transmission of phytopathogenic agents is as a major mean of dispersal and is therefore significant in the emergence of disease (Baker and Smith, 1966; Barret et al., 2016). Introduction of microorganism possessing plant-growth promoting or biocontrol activity into or on seed is a promising approach for increasing agricultural yield. For instance, the plant-growth promoting bacterial strain *Paraburkholderia phytofirmans* PsJN can be successfully inserted into seeds of several plant species through the floral pathway (Mitter et al., 2017). However, in other cases inoculation success is highly variable between seed samples (Bacilio-Jiménez et al., 2001). This inconsistency could be partly explained by the empirical selection of the biocontrol agents, which could be deficient regarding seedling colonization potential. Further knowledge about the nature, succession and activities of seed-borne microbial communities during germination and emergence could provide clues on the molecular basis of efficient seedling colonization.

Since in some ecosystems the “*assembly of bacterial community is based on functional genes rather than species*” (Burke et al., 2011), studying the functional repertoire of the seed microbiota is an interesting starting point for predicting the assembly of the plant microbiota during the first steps of plant life developmental stage. The aim of this study was therefore to uncover functional microbial traits that could be implicated in assembly of the plant microbiota at early stages of the plant cycle, namely seed germination and emergence. For this purpose, we analyzed the metagenomes of microbial assemblages associated to seeds, germinating seeds and seedlings of two different plant species: bean (*Phaseolus vulgaris*) and radish (*Raphanus sativus*). Phylogenetic classification of metagenomics reads highlighted significant changes in the composition of microbial assemblages during emergence with enrichment of bacterial related to *Enterobacteriales* and *Pseudomonadales*. Moreover, prediction of the functional profiles of these assemblages revealed bacterial traits potentially involved in the selection of these taxa in seedlings. In addition, we observed shifts in bacterial assemblages’ lifestyle during emergence at the composition and functional level. These shifts could be related to the enrichment of copiotrophic taxa over oliogotrophic taxa. Based on these data, we propose that selection during plant germination and emergence would favor bacteria species with fast-growing capabilities, in response to the increase in nutrient availability. All together, the data presented in this manuscript reveal novel insights about determinants involved in transmission of bacteria from seed to seedlings, therefore providing new perspectives for selection of seed inoculants.

## Materials and Methods

### Plant Species and Sample Preparation

Two commercial seed lots corresponding to bean (*Phaseolus vulgaris* var. Rocdor, Vilmorin) and radish (*Raphanus sativus* var. FH12, Vilmorin) were employed in this study. Three subsamples of 10,000 radish seeds and 1,000 bean seeds were used for DNA extraction. Radish and bean seeds were incubated in 2 ml of PBS supplemented with 0.05% Tween20 (Sigma–Aldrich Co. LLC) per gram of seed under constant agitation (150 rpm) at 4°C for 2 h30 and 16 h, respectively. Suspensions were centrifuged (12,000 × *g*, 20 min, 4°C) and pellet were stored at -80°C until DNA extraction. Germinating seeds and seedlings were obtained in axenic germination boxes using the protocol described in Barret et al. (2015) with some minor’s modifications. Briefly, 1,200 radish seeds and 600 bean seeds were placed in several axenic germination boxes. Germination boxes were incubated at 20°C at a photoperiod of 16 h light/8 h darkness. Germinating seeds were collected when visible protrusion of the radicle was observed. Seedlings were collected at appearance of the cotyledons. All experiments were independently repeated three times during three different weeks. Germinating seeds and seedlings were soaked in PBS supplemented with 0.05% Tween 20 and mixed softly with a Stomacher^®^ lab paddle blender during 2 min. Suspensions were centrifuged (12,000 × g, 20 min at 4°C) and pellets were stored at -80°C. Total genomic DNA was extracted with the Nucleospin Food kit (Macherey-Nagel, Duren, Germany) according to the manufacturer’s protocol with some small modifications in the lysis step (two-three volumes of the lysis buffer plus proteinase K were used depending of the biomass).

### Seed Exudates Analysis

Seed exudates were collected according to protocol described previously (Kageyama and Nelson, 2003) with some modifications. Three seed subsamples (12 g each) were used for exudates preparations. Seeds were surface sterilized through incubation for 10 min in a solution of sodium hypochlorite (1% available chlorine w/v) containing 0.05% Tween 20. Then, seeds were incubated twice in a solution of ethanol 90% during 5 min and rinsed three times in sterile water. Surface-disinfected seeds were soaked in 100 ml of sterile water and kept at 23°C on a rotary shaker overnight. After incubation, the solution was filtered sequentially through 100 μM and 0.22 μM filters (Whatman). The filtrate was freeze at -80°C, lyophilized and stored at -20°C. Exudate sugar composition was determinate by gas chromatography coupled to mass detection (GC-FID/MS) and exudate amino acid concentrations by reversed phase Ultra Performance Liquid Chromatography (UPLC) coupled to MS detection at the IGEPP’s Metabolic Profiling and Metabolomic Platform (P2M2) in Rennes (France).

### Shotgun Metagenomics Libraries Preparation

All DNA samples were sequenced at the Genome-Transcriptome core facilities (Genotoul GeT-PlaGe; France). DNA-sequencing libraries have been prepared according to Illumina’s protocols using the Illumina TruSeq Nano DNA LT Library Prep Kit. Library quality was assessed using an Advanced Analytical Fragment Analyser and libraries were quantified by QPCR using the Kapa Library Quantification Kit. DNA-sequencing experiments have been performed on an Illumina HiSeq3000 using a paired-end read length of 2 × 150 pb with the Illumina HiSeq3000 Reagent Kits.

### Trimming of Reads and Plant-Derived ORF of Plant Origin

Paired-end reads containing more than one ambiguity, bases with Qscore < 25 and Illumina adapters were removed with cutadapt v 1.8 (Martin, 2011). Plant-related reads were removed via mapping of the reads with BowTie2 v2.2.4 (Langmead and Salzberg, 2012) against representative genomic sequences of *Brassicaceae*-radish and bean. Based on this procedure approximately 25% of the reads were removed from the metagenomics datasets (Supplementary Table S1). Trimmed reads were then used for the meta-assembly, where ORF of plant origin were also examined. To crosscheck the presence of plant related genes in our dataset, we performed a BLASTn of all the contigs against two specific databases with different plant and plastid genomes, and ORFs with significant plant BLASTn hits were removed. After the trimming process, approximately 30% of the predicted ORF were from microbial origin and affiliated to one COG.

### Inferences of Phylogenetic and Functional Composition

The phylogenetic profile of each metagenomic sample was estimated with CLARK, a k-mer based classifier (Ounit et al., 2015). Reads were classified at the bacterial genus level using an in-house database containing 2,793 whole genome sequences. Classified metagenomic reads were used for community average size estimation (AGS) with the software MicrobeCensus (Hyatt et al., 2012). This software estimates AGS by aligning reads to a set of universal single-copy gene families present in almost all microorganisms.

A meta-assembly of all reads was performed with IDBA_UD (Peng et al., 2012), resulting in a non-redundant assembly of 947.5 Mb for bean reads and 822.5 Mb for radish. A gene catalog was created through ORF prediction with FragGeneScan v1.20 (Rho et al., 2010). Reads of each sample were mapped with BowTie2 against the gene catalog and converted to bam files with Samtools v1.2.1 (Li et al., 2009). Bam files were used to count the number of reads occurrence within each predicted ORF. Orthology inference of each ORF was performed by homology search (GHOSTZ v1.0) (Suzuki et al., 2014) against the EggNOG v4.0 database (Jensen et al., 2007).

### Metagenome Sequence Binning

To take maximal advantage of the differential coverage needed for the binning process, individual assemblies were performed with IDBA_UD for each metagenome dataset, resulting in a total of 18 distinct assemblies. All assemblies were merged in a unique meta-assembly file for each plant species. Reads were mapped to the meta-assembly files using Bowtie2 and converted to bam files. Mapped reads and meta-assemblies were used for an initial binning step with Metabat v.0.32.4 (Kang et al., 2015) (Supplementary Table S2). Bin completeness, contamination and strain heterogeneity were evaluated with CheckM v 1.0.4 (Parks et al., 2015). Contamination indicates the percentage of specific marker genes that are found in multiple copy number, and strain heterogeneity values indicate the percentage of multi-copy marker genes with more than 90% identity. Bins with a minimum of 60% completeness were selected for subsequent analyses. Since individual assemblies were merged to a single meta-assembly per plant species, some duplicated contigs were present in the bins. Hence, duplicated bins (100% identity) were removed with NUCmer v 3.1 (Kurtz et al., 2004) and only the largest contig was conserved in the genome bin. A total of 27 bins with >70% completeness and up to 96% contamination were selected for further processing (Supplementary Table S3). For improving bin specificity (decrease the rate of contamination), genomic bins were represented using the R package *mmgenome* (Karst et al., 2016) (Supplementary Figure S1). At the end of the filtering process, we managed to reconstruct 19 high quality draft metagenome-assembled genomes (MAGs) from bean and radish metagenomes (**Table 1**). To have a first glimpse of the phylogenetic affiliation of each MAG, a BLASTn search of the first 5000 nt of the 10 largest contigs MAGs was performed against the NCBI nt database. Then representative genomic sequences of each taxa were retrieved from the NCBI to calculate Average Nucleotide Identity based on BLAST (ANIb) with Jspecies. Closest ANIb values were selected for references genomes and used for Circle Packing representation with the D3.js JavaScript library (Supplementary Table S4). Circle Packing and ANIb were calculated in the galaxy interface of the French collection of Plant Associated Bacteria^1^.

**Table 1:** General statistics of the 19 draft metagenome-assembled genomes (MAGs) reconstructed from bean and radish metagenomes.

MAG	Completeness	Contamination	Strain heterogeneity	# Contigs	Size (Mb)	Coverage			Phylogenetic affiliation
							
						S	G	P	
MAGb21	100	0	0	43	3.96	15.62	0.01	0.01	*Acinetobacter* sp like
MAGb07	97.32	0.08	0	52	5.94	161.36	8.90	20.63	*Pseudomonas moraviensis*
MAGr09	86.84	23.74	95.38	90	3.33	23.26	0.31	0.99	*Enterobacteriales*
MAGb17	99.29	1.32	14.29	87	4.65	31.85	1.23	12.65	*Rosenbergiella nectarea*
MAGb15	98.79	0.51	16.67	56	4.96	1.62	35.43	19.82	*Pseudomonas fulva*
MAGb14	94.3	0.98	80	110	4.58	1.64	32.44	21.81	*Pseudomonas fulva*
MAGb12	92.47	2.27	22.73	497	5.46	3.11	13.70	3.68	*Pseudomonas* sp. like
MAGb24	93.44	0.79	14.29	36	3.12	0.79	24.51	5.50	*Rosenbergiella nectarea*
MAGb18	90.07	29.4	95.56	843	3.91	0.31	19.75	2.01	*Bacillus* sp. like
MAGb13	98.03	1.41	12.5	120	5.21	0.52	35.15	5.20	*Enterobacteriales*
MAGb04	93.22	0.17	33.33	33	4.3	4.74	57.79	87.51	*Pseudomonas* sp. *RIT-Pia*
MAGb09	98.57	15.19	87.9	278	5.83	0.67	0.87	110.52	*Pseudomonas psychrotolerans* sp. like
MAGb05	72.28	14.04	100	110	4.7	3.47	1.04	25.59	*Pseudomonas graminis* sp. like
MAGb03	98.03	0.08	0	47	4.03	138.30	153.24	1683.91	*Pantoea agglomerans*
MAGr08	86.14	23.67	100	260	3.97	5.66	0.22	32.6	*Erwinia gerundiensis*
MAGr14	91.7	4.1	19.57	377	4.24	0.6	0.12	14.71	*Pantoea sp. IMH*
MAGb26	72.72	0.08	0	64	2.62	1.90	2.38	68.37	*Erwinia gerundiensis*
MAGb19	96.58	0.72	50	79	4.2	0.69	1.45	32.77	*Leclercia adecarboxylata* sp. like
MAGr12	97.64	1.32	16.67	236	5.18	0.03	0	10.85	*Flavobacterium* sp. like


*Completeness, contamination and strain heterogenity is based on CheckM v 1.0.4. Contamination indicate the percentage of specific marker genes that are found in multiple copy number, and strain heterogeneity values indicate the percentage of multi-copy marker genes with more than 90% sequence identity. MAG coverage was calculated with their corresponding reads (either bean or radish). Thus, coverage (C) was computed with the formula C = LN/B, where L stands for read length in bp (150), N stands for number of reads mapping the MAG and B is the total length of the MAG in bp. S, seeds; G, Germinating seeds; P, Seedlings.*

### Statistical Analysis

To analyze bacterial community composition between plant developmental stages, we used a normalization procedure related to the proportion. Genera with a frequency higher than 0.1% were selected to avoid the presence of false positive. Statistical analyses were done with Rstudio v3.1 using with the R package *phyloseq* (McMurdie et al., 2013). Observed genus/COG richness (number of genus) and evenness (estimated with Simpson’s reciprocal index) were calculated on genus and COG tables rarefied to 100,000 sequences and 10,000 COG per sample respectively. Differences in richness and evenness between variables were assessed by one-way ANOVA with post-hoc Tukey’s HSD test. Variances in community membership and composition between plant developmental stages were assessed with Jaccard and Bray–Curtis indices, respectively. Jaccard and Bray–Curtis indices were calculated on genus/COG table transformed to even sampling depth. Model of multivariate analysis of variance was constructed using based on the Bray–Curtis distance using the function *Capscale* of the R package *vegan* 2.4.2 to determine the most influential environmental variables on the bacterial community composition.

Principal coordinate analysis (PCoA) was used for ordination of Jaccard and Bray–Curtis distances. Permutational multivariate analysis of variance (PERMANOVA) was carried out to assess the importance of the plant developmental stage on microbial community profiles. To quantify the contribution of the plant generation in microbial community profiles, canonical analysis of principal coordinates was performed with the function *Capscale* of the R package *vegan* 2.4.2 followed with PERMANOVA with the function *adonis*. Changes in relative abundance of bacterial genera/COG between the different plant developmental stages (seeds, germinating seeds and seedlings) were assessed with likelihood ratio test (LRT) with the R package *DESeq2 1.14.1* (Anders and Huber, 2010). The different *rrn* copy number of enriched or depleted taxa were calculated by using the *rrn*DB database (Stoddard et al., 2015).

### Growth Potential of Seed-Associated Bacterial Isolates

Nine bacteria strains from radish and bean seed samples were isolated on 1/10 strength Tryptic Soy Agar (TSA; 17 g.l^-1^ tryptone, 3 g.l^-1^ soybean peptone, 2.5 g.l^-1^ glucose, 5 g.l^-1^ NaCl, 5 g.l^-1^ K2HPO4, and 15 g.l^-1^ agar) after 7 days of incubation at 18°C. The isolates were typed through sequencing of a portion of *gyr*B with the primer set gyrB_aF64 and gyrB_aR353 following the procedure described in Barret et al. (2015). These isolates were chosen because they represent the main taxa enriched or depleted in seedlings (identified with the R package *DESeq*2 1.14). We next tested the growth of these isolates on bean and radish seed exudates. Bacterial inocula were grown for 48 h on 1/10 strength TSA media. Then, bacterial cells were scraped from agar plates, suspended in sterile distilled water and calibrated to 1 × 10^8^ CFU ml^-1^. These bacteria inocula were incubated in radish and bean exudates during 48h with absorbance measures every 2 h. Absorbance data was fit to the standard form of the growth curve logistic equation by using the Rpackage *Growthcurve* (Sprouffske and Wagner, 2016). We used the area under the curve (optical density [OD] in time) as a proxy for assessing growth of the different bacteria strains (Herrera Paredes et al., 2018).

### Data Access

The datasets generated and analyzed during the current study are available in the European Molecular Biology Laboratory (EMBL)-European Bioinformatics (EBI) under accession no. PRJEB22845. Tables and scripts used in this work are public available in GitHub^2^.

## Results

Changes in phylogenetic and functional composition of the seed microbiota during germination and emergence were assessed in two distinct plant species, namely common bean (*Phaseolus vulgaris*) and radish (*Raphanus sativus*). Nine samples corresponding to seeds (3), germinating-seeds (3) and seedlings (3) were obtained for each plant species. DNA extraction and subsequent Illumina HiSeq3000 sequencing were performed on these 18 samples. After quality-filtering (see experimental procedure), a total of 57.5 Gb were obtained, with an average of 21.3 million paired-end reads per sample (Supplementary Table S1).

### Emergence Shapes the Structure of the Seed Microbiota and Selects Certain Bacteria Taxa

**FIGURE 1:**
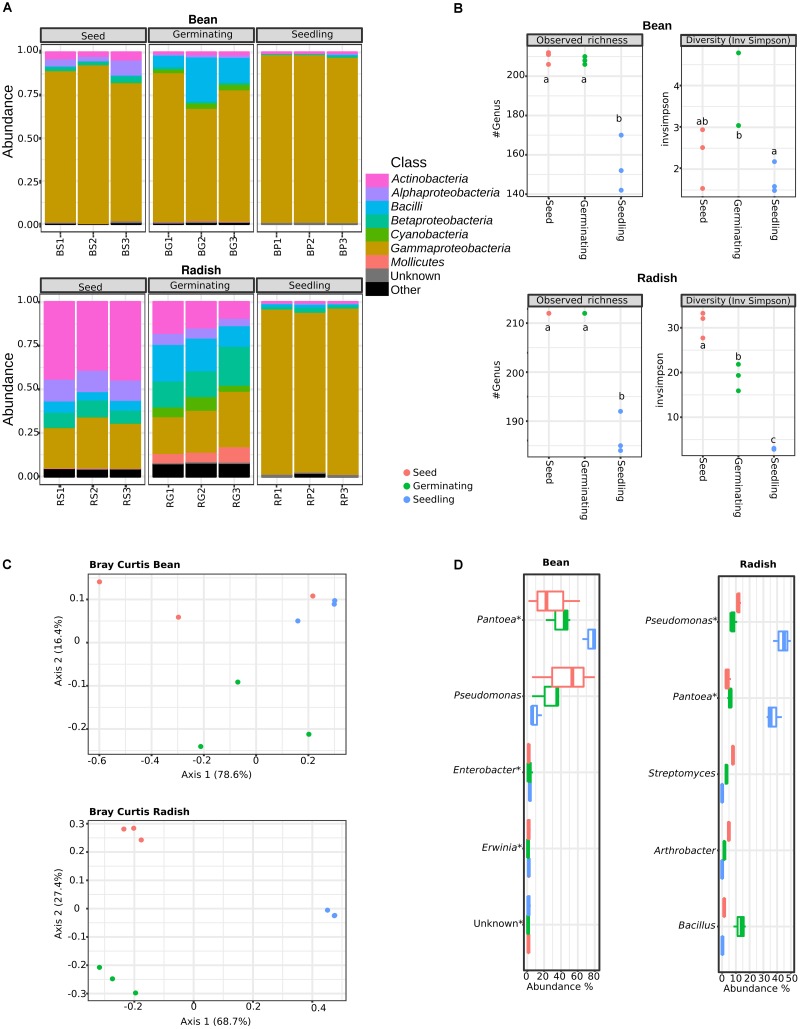
Structure and composition of microbial assemblages associated to seeds, germinating seeds and seedlings. **(A)** Phylogenetic assignment of reads in bacterial classes was performed with Clark, a k-mer based approach. **(B)** Changes in microbial richness (left panels) and diversity (right panels) among the samples investigated. Letters indicate differences between individual means as assessed with one factor ANOVA with *post hoc* Tukey HSD (*P* < 0.01). **(C)** PCoA plots based on Bray–Curtis index calculated at the genus level. **(D)** Abundant bacterial genus in the samples investigated. Asterisks (^∗^) indicate taxa that are significantly more abundant in seedling samples in comparison to seeds (DeSeq2; log2 FC > 3, *P* < 0.01). S, seeds; G, germinating seeds and P: Seedlings.

The phylogenetic composition of bacterial assemblages was predicted with a k-mer based reads classification method. On average, 23 and 9% of all reads were assigned at the bacterial genus level for bean and radish, respectively (Supplementary Table S1). Based on this phylogenetic inference, bacterial assemblages associated to bean seeds were mainly composed of *Gammaproteobacteria*, while bacterial assemblages of radish seeds were more diverse and dominated by *Actinobacteria*, *Gammaproteobacteria* and *Alphaproteobacteria* (**Figure 1A**). This higher bacterial diversity in radish seeds was also observed at the genus level with a ten-fold increase of reciprocal Simpson’s index in radish in comparison to bean (**Figure 1B** and Supplementary Figure S2). One of the factors that may explain the higher estimated diversity in radish could be the superior carrying capacity (i.e., number of individuals that the habitat can support) (McArthur, 2006) of radish seed in comparison to bean seed.

The dynamics of seed-associate bacterial assemblages during germination and emergence was investigated on germinating-seeds and seedlings. According to CLARK classification, a significant decline of genus richness was observed in bean and radish during emergence (one-way ANOVA with *post hoc* Tukey HSD, *P* < 0.001, **Figure 1B**). This decrease in richness is associated with a marked increase in the relative abundance of *Gammaproteobacteria* (**Figure 1A**), thereby confirming previous community profiling surveys performed with 16S rRNA gene and *gyrB* on a range of plant species belonging to *Brassicaceae* and *Fabaceae* (Barret et al., 2015).

Similarity in community composition between seed, germinating seeds and seedlings was subsequently estimated through ordination of Bray–Curtis index with PCoA (**Figure 1C**). The relative contribution of the plant developmental stage in community profiles was further investigated through canonical analysis of principal coordinates (CAP) followed by PERMANOVA. According to CAP analyses, the plant developmental stage explained 95% (*p* = 0.0036) and 69.5% (*p* = 0.0107) of variation in bacterial community composition in radish and bean samples, respectively. Shift in bean bacterial assemblage composition during germination and emergence was less pronounced than radish probably as a result of a lower initial bacterial diversity in bean seeds (**Figure 1B**).

To identify bacterial genera selected at the early plant developmental stages, differences in genera relative abundance were assessed with DeSeq2. Overall, 30 and 43 bacterial genera were significantly (log2 FC > 3; *P* < 0.01) enriched in bean and radish seedlings, respectively (**Figure 1D** and Supplementary Figure S3). Most of these enriched genera were associated to the *Enterobacteriaceae* and *Erwiniaceae* fam. nov. (Adeolu et al., 2016) and comprises taxa belonging to *Enterobacter* and *Pantoea* that have been frequently associated to the spermosphere of various plant species (Nelson, 2017). Taxa significantly (log2 FC > 3; *P* < 0.01) depleted in bean and radish seedlings correspond to bacteria belonging to the *Firmicutes* and *Actinobacteria* groups. The presence of a number of potential human pathogenic bacteria enriched in seedlings (Supplementary Figure S3) such as *Salmonella* or *Yersinia* have to be interpret with cautions since it could be related to reads falsely assigned to these genera as a result of their phylogenetic proximity with other members of the *Enterobacteriaceae* and *Erwiniaceae.* In addition to bacterial taxa belonging to the *Enterobacteriales*, the *Pseudomonas* genus is also significantly enriched in radish seedlings, but not in bean seedlings (**Figure 1D**).

Overall, analyses of the phylogenetic composition of plant-associated microbial assemblages during germination and emergence show that emergence shapes the structure of the seed-associated bacterial community and selects a group of taxa belonging to the *Gammaproteobacteria*, which is probably an indication of a strong selective force of the young plant on seed-borne microorganisms.

### Seed Germination and Emergence Select a Core of Microbial Functions

Analysis of the phylogenetic composition of seed-associated bacterial assemblages revealed that some bacterial taxa belonging to *Gammaproteobacteria* are selected during emergence. To identify the bacterial determinants involved in this selection, we predicted the functional composition of the metagenomics samples through annotation of a gene repertoire. A total of 22,059 and 22,116 cluster of orthologous groups (COGs) were obtained for all bean and radish samples, respectively.

**FIGURE 2:**
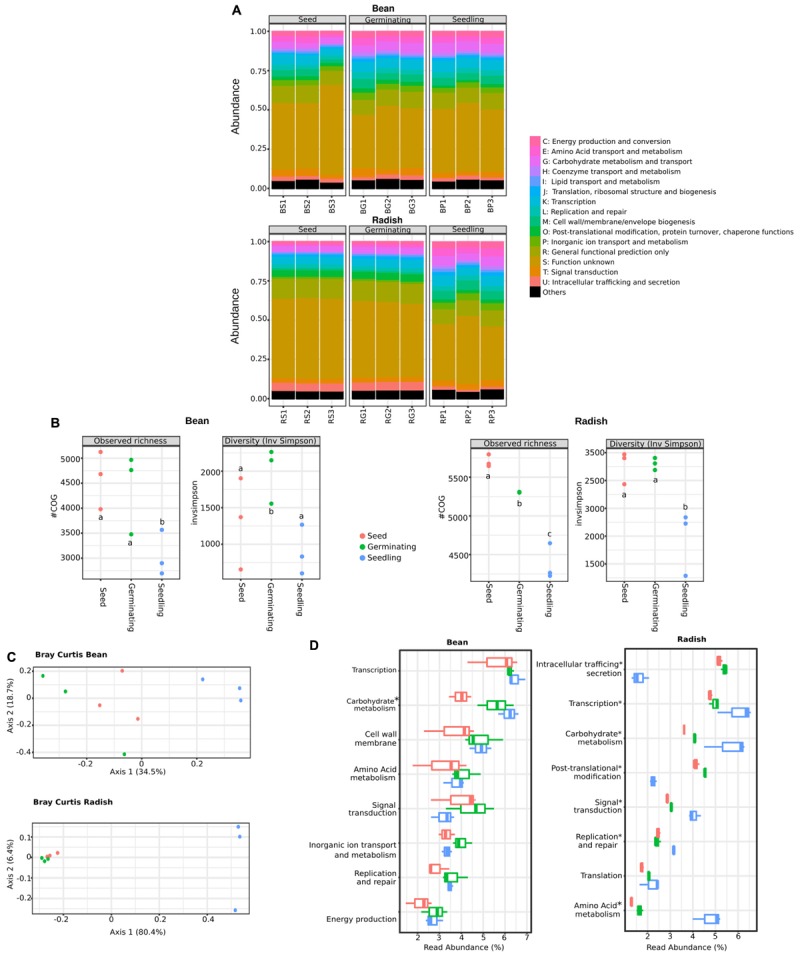
Functional composition of microbial assemblages associated to seeds, germinating seeds and seedlings. **(A)** Relative abundance of COGs functional categories in the predicted proteomes of the samples investigated. **(B)** Changes in COGs richness (left panels) and diversity (right panels) among the samples investigated. Letters indicate differences between individual means as assessed with one factor ANOVA with *post hoc* Tukey HSD (*P* < 0.01). **(C)** PCoA plots based on Bray-Curtis index calculated on COGs table. **(D)** Abundant functional categories in the samples investigated. Of note is that the functional categories S (function unknown) and R (general functional prediction only) were not reported in this panel. Asterisks (^∗^) indicate taxa that are significantly more abundant in seedling samples in comparison to seeds [ANOVA with *post hoc* Tukey HSD (*P* < 0.05)].

According to COG functional categories, the functional profiles of metagenomics samples were conserved between plant species (**Figure 2A**). Thus, despite variation in phylogenetic profiles, broad functional categories are conserved in the different assemblages therefore suggesting functional redundancy between members of these assemblages (Allison and Martiny, 2008). At a fine-grained description (COG level), we observed a significant decrease (one-way ANOVA with *post hoc* Tukey HSD, *P* < 0.01) of COG richness in radish and bean seedlings in comparison to seeds and germinating seeds (**Figure 2B**). A significant decrease of COG diversity was also noted during emergence of radish (**Figure 2B**). Comparison of functional community composition between microbial assemblages revealed a significant clustering of seedling samples for both plant species (**Figure 2C**) (PERMANOVA, *P* = 0.007 and *P* = 0.002 for bean and radish, respectively). Therefore, emergence is not only affecting phylogenetic bacterial diversity but also functional diversity.

To evaluate the resistance of the seed-associated microbial assemblages to soil or air-borne microbial invaders, we calculated multifunctional redundancy (MR) (Miki et al., 2013) in our samples. Thus, the estimated taxonomic (with Shannon’s diversity indexes calculated on bacterial genera) and the functional diversities (with COGs) were calculated in seeds, germinating seeds and seedlings (Supplementary Figure S4). The pattern of the relationship between taxonomic and functional diversities illustrate the degree of MR within an assemblage, with a linear relationship being indicative of weak functional redundancy (Peter and Sommaruga, 2016). Hence, the linear relationship between structure and function of bean-associated microbial assemblages suggested a limited MR that would be then prone to invasion by alien species at the seedling stage (Supplementary Figure S4). In contrast, the more saturating linear relationship between structure and function of radish-associated microbial assemblages implies that these assemblages would be more resistant to invasion. These data are an interesting starting point to evaluate the resistance of seed-associated microbial assemblages although additional inoculations experiments need to be done to evaluate it.

Moreover, a process-centric approach (Tringe et al., 2005) was used to highlight bacterial functions significantly enriched in seedling metagenomes in comparison to seed metagenomes. With such an approach, COGs overrepresented in seedlings are expected to be selected by the local environment and could then provide an important function in this habitat. A total of 541 and 86 COGs (Supplementary Table S5) were significantly enriched in bean and radish seedlings in comparison to seeds (log2FC > 5, *P* < 0.01). Only 6 COGs were enriched in seedlings of bean and radish, therefore suggesting differences in response of microbial assemblages to emergence of distinct plant species. However, at functional level, we identified genotypic features of seed-colonizing bacteria enriched in seedlings in both plant species, like seedling-associated enrichment of genes linked to chemotaxis and adhesion (methyl-accepting chemotaxis, extracellular solute-binding protein, for example), signal transduction mechanism (histidine kinase, response and transcriptional regulators) or motility (via flagella or type IV secretion system). Together, these genes functions are likely involved in the colonization and intimate association of the microorganism with the plant host. In addition, at the broad functional level, we found numerous COGs related to carbohydrate metabolism enriched in both germinating seeds and seedlings of bean and radish (**Figure 2D**). Moreover, COGs associated to amino acid metabolism, transcription and signal transduction were increased in radish seedlings. Altogether, this COG enrichment suggests that the shift of bacterial composition observed in seedlings could partly be explained by the superior growing abilities on exudates release during germination of selected taxa. Analysis of the nature of seed exudates produced under experimental conditions indicated that monosaccharide (glucose and fructose), disaccharide (sucrose) and amino acids (alanine, asparagine) are released during germination (Supplementary Figure S5), which is in accordance with previous analyses of seed exudates performed on a range of plant species (Nelson, 2004; Schiltz et al., 2015). Thus, the composition of the seed exudates together with the enrichment of COG associated to their metabolism suggest that the taxa enriched during emergence are the ones able to grow faster in this medium.

### Shifts Toward a Bacterial Copiotrophic Life Strategy in Seedlings

Enrichment in the relative abundance of some bacterial taxa and functional traits in seedlings could be due to the responsiveness of these taxa to an increase of carbon sources availability occurring during germination, a physiological trait known as copiotrophy (Ho et al., 2017). Since the combination of genome size and *rrn* copy number in bacterial taxa can be used as a marker of high growth rate (copiotrophy) and versatility (Cobo-Simón and Tamames, 2017), we decided to investigate these features in the annotated metagenomes datasets.

**FIGURE 3:**
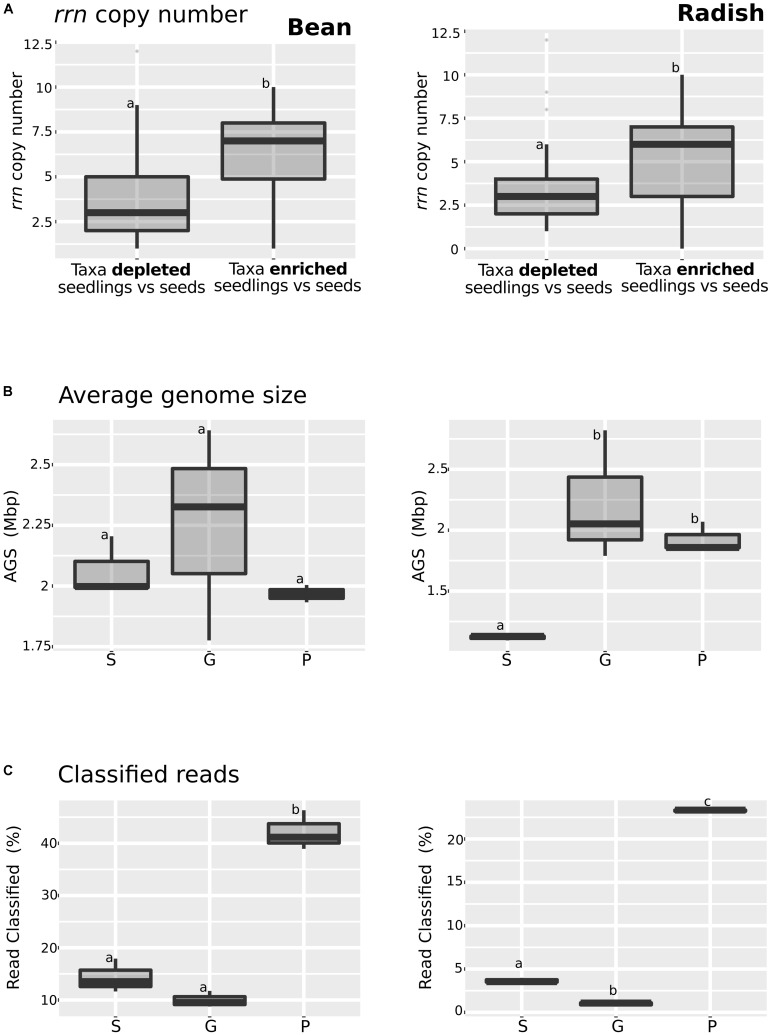
Analysis of some of the key copiotrophic characteristics in metagenome datasets. **(A)** *rrn* copy number of taxa that are significantly more or less abundant in seedling samples in comparison to seeds (DeSeq2; log2 FC > 3, *P* < 0.01). **(B)** Average genome size and **(C)** Proportion of classified reads among the samples investigated. Letters indicate differences between individual means assessed with one factor ANOVA with *post hoc* Tukey HSD (*P* < 0.05). The left part of the panel shows bean samples and the right part radish samples. S, seeds; G, germinating seeds, and P, Seedlings.

Bacterial taxa significantly enriched or depleted in seedlings in comparison to seeds were already identified using DeSeq2 (Supplementary Figure S3). To study *rrn* copy number in these taxa, we analyzed the described number of copies of the *rrn* in the *rrn*DB database (Stoddard et al., 2015) (**Figure 3A** and Supplementary Table S6) for each genus. This analysis showed that taxa that are enriched in seedlings have a significant (one-way ANOVA, *P* < 0.01) higher *rrn* copy number in both plant species. Next we estimated the genome size of members of microbial assemblages by using MicrobeCensus (Nayfach and Pollard, 2015). According to this prediction, the average genome size of microbial assemblage was constant in bean (**Figure 3B** and Supplementary Table S7). In contrast, a significant increase (one-way ANOVA, *P* < 0.05) in the average genome size of radish-associated microbial assemblage was observed on germinating seeds and seedlings, supporting the hypothesis of an enrichment of copiotrophic taxa with higher *rrn* copy number and larger genomes during plant emergence in radish (**Figure 3B**). Finally, we analyzed the proportion of CLARK classified reads (**Figure 3C**). We found a significantly increase of classified reads in seedlings in comparison to seeds and germinating seeds in both plant species. This increase of classified reads in seedlings is probably due to the overrepresentation of copiotrophic bacteria in seedling, which are easier to culture, and are thus likely very common in genomic databases (Leff et al., 2015).

**FIGURE 4:**
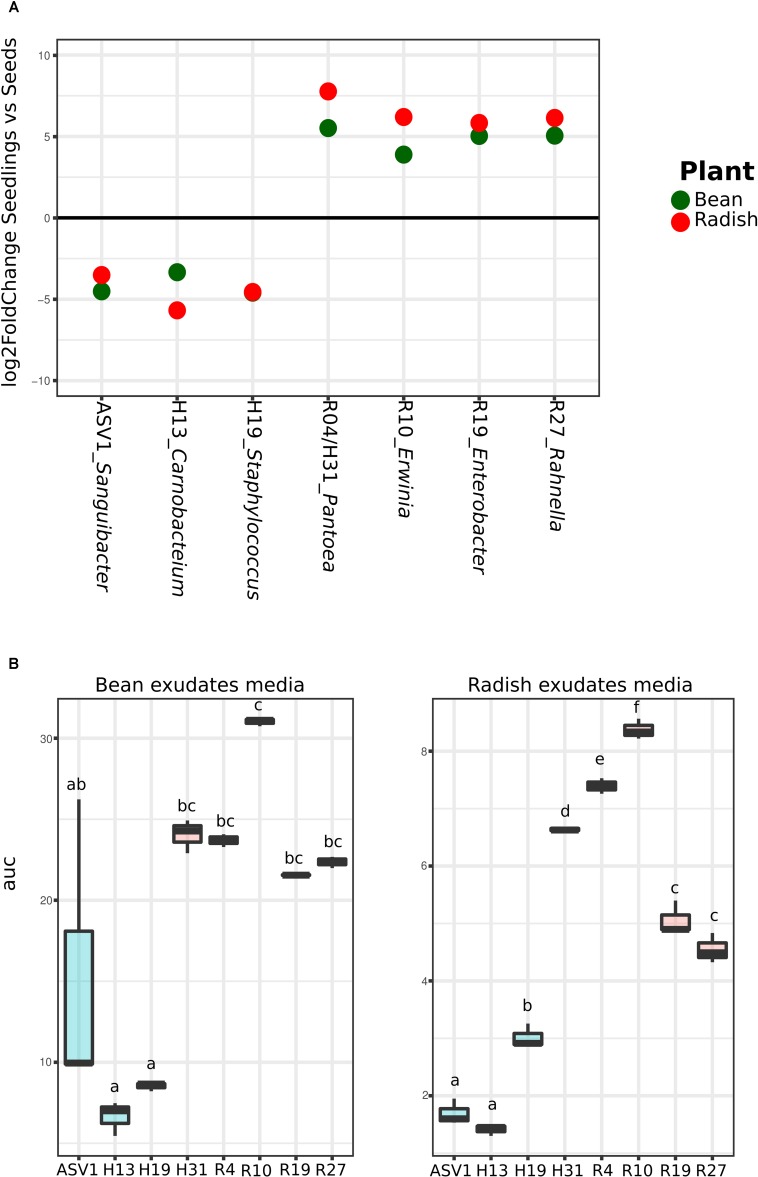
Quantification of seed-associated bacteria potential growth on bean and radish seed exudates. **(A)** Metagenomics reads relative abundance Log2fold change in seedlings in comparison to seeds of the nine selected taxa according to DeSeq2. **(B)** Area under the growth curve (optical density [OD] in time) as a marker of the potential growth of the different bacteria strains. Letters indicate differences between individual means assessed with one factor ANOVA with *post hoc* Tukey HSD (*P* < 0.05).

Analysis of the metagenomics data shows an enrichment of taxa with higher *rrn* copy number and larger genomes during plant emergence, suggesting that selection during plant emergence favors species with fast-growing capabilities. To test this proposal, nine bacteria strains were isolated from radish and bean seeds and typed with a portion of *gyr*B. These strains correspond to *Pantoea agglomerans* (R4 and H31), *Erwinia* sp. (R10), *Enterobacter* sp. (R19), *Rahnella* sp. (R27), *Staphylococcus* sp. (H19), *Carnobacter* sp. (H13), and *Sanguibacter* sp. (ASV1). These isolates were chosen because they represent the main taxa enriched or depleted in seedlings (log2 FC > 3; *P* < 0.01; identified with DESeq2 1.14). The strains R04, R10, R19, R27 and H31 belong to the main bacterial genus enriched in seedlings (*Pantoea*, *Erwinia*, *Enterobacter*, and *Rahnella*) whereas the strains H19, H13 and ASV1 belong to the main bacterial genus depleted in seedlings (*Staphylococcus*, *Carnobacter* and *Sanguibacter*) in both plant species (**Figure 4A** and Supplementary Table S6). Areas under the growth curves show that representative isolates selected in seedlings (R04, R10, R19, R27, and H31) have a greater growth potential than the representative isolates excluded in seedlings (H19, H13, and ASV1) (**Figure 4B**). Taking all together, the detail analysis of the metagenomic data and the quantification of bacterial growth capacity suggest a positive correlation between traits associated with copiotrophy and plant emergence.

### Genome Reconstruction of Bacterial Seed Colonizers

To gain a genome-wide view of the major bacterial populations associated to seed, germinating seeds and seedlings, contigs were clustered into MAGs with MetaBAT. According to the presence of a minimal set of essential genes, 19 MAGs with a completion > 70% and a contamination lower than 25% were obtained (**Table 1**). Fifteen of these MAGs could be classified as “high quality draft MAG” following the contamination and completion criteria described in Bowers et al. (2017). However, it must be noted that due to the multi-copy nature of the *rrn* operon, 5S, 16S and 23S rRNA, genes are not detected in any MAGs.

**FIGURE 5:**
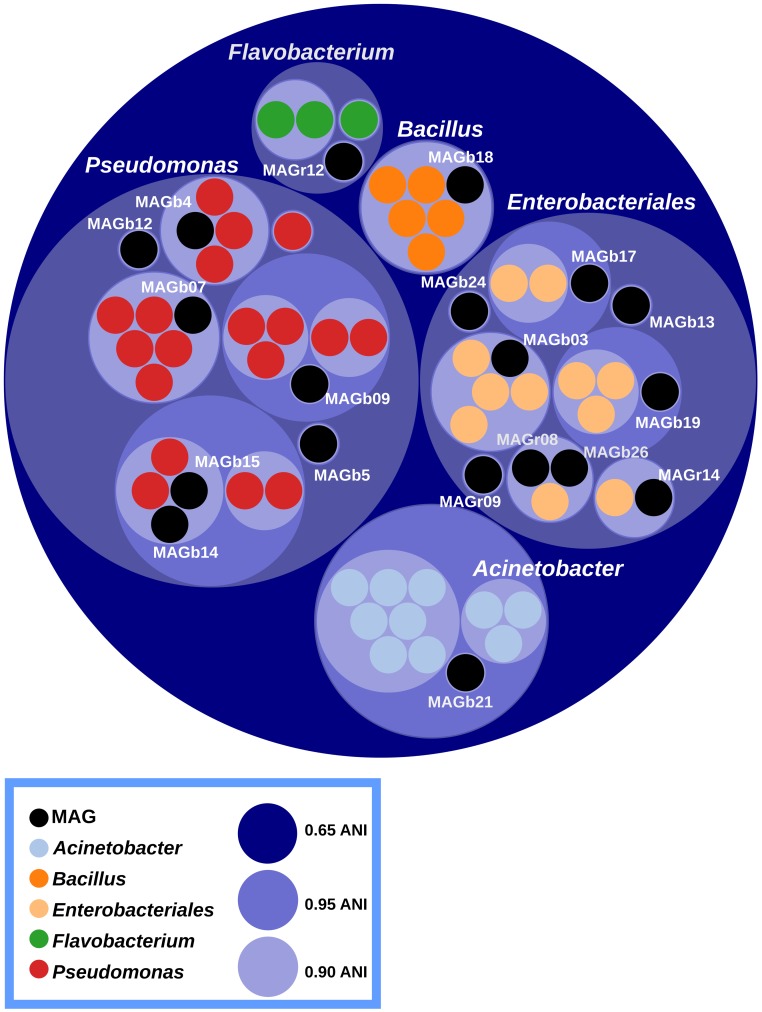
Circle packing visualization of ANIb values. MAGs (black circles) and closely related genome sequences (colors circles) have been clustered according to their ANIb values. Inner and outer circles represent ANIb values at a cutoff of 0.65, 0.90 and 0.95 respectively. Although, *Acinetobacter* belong to the *Pseudomonadales* order, the ANIb cut-off chosen placed the genus outside this order. A total of 10 MAGs could be considered as new bacterial cliques.

According to Average Nucleotide Identity based on blast (ANIb) values, the vast majority of MAGs (16 out of 19) belong to the two major bacterial orders found in seedling, namely *Pseudomonadales* and *Enterobacteriales* (**Figure 5** and Supplementary Figure S6). The two remaining MAGs belongs to the *Acinetobacter*, *Flavobacterium*, and *Bacillus* genera (**Figure 5**). Ten MAGs are representative genomes of new bacterial cliques since they their ANIb values are below 95%, a gold-standard for circumscribing prokaryotic species (Richter and Rossello-Mora, 2009). Among these new cliques, MAGb24 is affiliated to *Rosenbergiella nectarea*, a new enterobacterial species isolated from nectar of different plant species (Halpern et al., 2013).

To assess the dynamics of the bacterial populations during germination and emergence, we calculated the average coverage of the MAGs at each plant developmental stage. Based on this average coverage, we were able to identify eight MAGs enriched in seedlings (**Table 1**). With the exception of one MAG related to *Flavobacterium*, the other MAGs were all related to *Enterobacterales* and *Pseudomonadales*. Since other MAGs affiliated to these two bacterial orders were prevalent at other stages of the plant life cycle (seed and germinating seeds), COGs specifically encoded in seedling-associated MAGs seedling may represent bacterial determinants selected by the host plant. A total of 13 and 15 COGs were specifically found in seedling-associated MAGs affiliated to *Enterobacterales* and *Pseudomonadales*, respectively (Supplementary Figures S7, S8 and Supplementary Table S8). These COGs were related to general metabolic processes (Supplementary Table S8) and include genes associated to carbohydrate metabolism (e.g., sugar ABC transporter or 4-alpha-glucanotransferase). These observations have, however, to be interpreted with cautions since these specific COGs of seedling bacterial populations are linked to conserved bacterial function. Although broad annotation of the MAGs with COGs is an interesting starting point for the identification of key bacterial function present in these draft population genomes, a more comprehensive analysis based of signature of selection such as dN/dS (Bulgarelli et al., 2013) will be necessary for isolating bacterial genes specifically selected by the host during the early stages of its life cycle.

## Discussion

Seeds represent the initial microbial inoculum of plant microbiota and could subsequently have a significant impact on plant health and productivity. Up to date, the structure of the seed microbiota has been mainly estimated through traditional culture-dependent methods or community-profiling approaches, and therefore the functional potential of these microbial assemblages remain largely unknown. In this work, we performed a comprehensive analysis of the structure and function of the seed microbiota of two plant species, namely bean and radish, and investigated the dynamics of these assemblages during germination and emergence.

According to kmer-based reads classification, the seed microbiota of bean and radish were mainly composed of *Proteobacteria* and *Actinobacteria.* These phyla have been frequently associated with seeds of various plant species (Barret et al., 2015; Midha et al., 2016; Rybakova et al., 2017) and more generally to other plant habitats such as the phyllosphere and the rhizophere (Müller et al., 2016). At a finer taxonomic resolution, *Gammaproteobacteria* are the main bacterial class associated to bean seeds, while radish seeds are more diverse, being composed of *Actinobacteria*, *Alpha*-, *Beta* and *Gamma-proteobacteria*. Although these taxa are common inhabitants of the seed microbiota, their relative abundances can vary significantly between seed samples of the same plant species (Barret et al., 2015; Klaedtke et al., 2016; Rezki et al., 2017; Rybakova et al., 2017). These variations in community composition could be driven by the plant genotypes, but also by abiotic factors, such as field management practices, harvesting methods, seed processing and storage (Barret et al., 2015). Indeed, it has been already shown that the production region could drive the composition of the seed fungal communities (Klaedtke et al., 2016). In addition, we cannot exclude the possibility of the observed variations in the community composition being a result of the inherent differences between amplicon-based and shotgun metagenomics-based approaches. Whereas seed microbiota studies made so far are amplicon-based (which could have different bias due to the PCR amplification, for example), the taxonomy classification presented here is built upon reference-free methods based on the analysis of the oligonucleotides (k-mers) frequency, previously applied to reference genomes. A limiting factor for this methodology is the availability of proper reference genomes to compare with.

Independently on the initial composition of the seed microbiota, germination affects microbial diversity in both plants tested. During plant germination, seeds shift from a quiescent state to an active physiological state where numerous substances are released into the surrounding environment (Nelson, 2004). As a consequence, microorganisms could persist within or on seeds as dormant cells and exit dormancy during germination by using these released nutrients. Although this hypothesis has to be validated experimentally, indirect evidence suggests that some bacteria members exit dormancy during plant germination. For instance, the increase in the number of reads affiliated to *Bacilli* on germinating seeds could correspond to germination of spore-forming bacterial in response to variation in environmental cues (e.g., nutrient or water availability) (Nelson, 2017). Under our experimental conditions, this increase in the relative abundance of *Bacilli* is transient and, during emergence, they are probably outcompeted by other *Gammaproteobacteria* species. Indeed, *Gammaproteobacteria* becomes the most abundant bacterial class associated to seedlings, as it was already observed in barley (Yang et al., 2017) and in different *Fabaceae* and *Brassicaceae* species (Barret et al., 2015). Since plant defense responses are not expected to be induced during plant emergence (Darrasse et al., 2010), it is tempting to speculate that this high prevalence of *Gammaproteobacteria* in seedlings is the result of fitness differences between these taxa and other members of the seed microbiota during plant emergence.

To gain insights into the bacterial functional determinants selected during germination and emergence, we analyzed the functional profiles of all metagenomics datasets. Significant differences in COG composition were observed between seed, germinating seed and seedling samples for both plant species. Interestingly, we observed an enrichment of COGs involved in carbohydrate or amino acid metabolism during emergence. The effective seed to seedling transmission of bacterial populations could be therefore related to carbohydrate and amino acids catabolism, as it has been previously shown for *Enterobacter cloacae* during the spermosphere colonization of multiple plant species (Nelson, 2017). Therefore, one may argue that copiotrophic microorganisms, capable of rapid growth in response to an increase in nutrient availability, are selected during emergence. To test this hypothesis, we have investigated in details different copiotrophic functional traits like *rrn* copy numbers or genome sizes (Lauro et al., 2009; Giovannoni et al., 2014; Roller et al., 2016). We found that the taxa enriched in seedlings have a higher *rrn* copy number and that the genomes present in seedling metagenomes are significantly larger than those present in seeds and germinating seeds. Moreover, the quantification of bacterial growth capacity of taxa enriched and depleted in seedlings show that representative isolates selected in seedlings have a greater growth potential than the representative isolates excluded in seedlings. Together, these results suggest that nutrient availability during emergence may produce a change toward a bacterial copiotrophic life strategy in seed-borne microorganisms. Remarkably, genes linked to chemotaxis and adhesion, signal transduction or motility were significantly enriched in seedlings as well. Many of these features have been previously and individually implicated in bacterial plant colonization in different cultivated microorganisms (Scharf et al., 2016). However, due to the fact that the main taxa selected in seedlings belong to the *Proteobacteria* group whereas taxa excluded in seedlings belong mainly to the *Actinobacteria* and *Firmicutes* groups, we cannot rule out completely the possibility of the difference in growth rate being also related to phylogeny and not exclusively to physiology.

The decrease of the phylogenetic and functional diversity observed during emergence under our soilless experimental conditions is probably counterbalanced in nature by acquisition of soil-borne microorganisms in the spermosphere. For instance, it has been previously shown that surface-sterilized rice seedlings are colonized by soil-borne microorganisms 24 h after seedling transplantation (Edwards et al., 2015). While the relative importance of vertical versus horizontal transmission in the assembly of the plant microbiota is currently unknown (Shade et al., 2017), we can predict the resistance of resident microbial communities (seed-borne) to invaders (air-borne; soil-borne) by calculating their multifunctional diversity. In bean, accumulation curves of multifunctional diversity suggested a limited buffering capacity that is therefore prone to invasion by migrants. In contrast, radish samples displayed a greater MR, therefore suggesting that radish seed bacterial communities would be more resistant against the invasion of new microbial colonizers in the spermosphere. Rybakova et al. (2017) also established, by analyzing bacterial networks, that the microbial community in another *Brassicaceae* species, namely rapeseed, is especially tight and probably resistant to invaders. These results are an interesting starting point for future inoculation experiments to validate this hypothesis.

The fate of seed-borne microorganisms during the plant life’s cycle and their roles in the assembly of the adult plant microbiota remains to be assessed. Although the composition of the plant microbiota is changing throughout the development of the host (Chaparro et al., 2014), we managed to reconstruct high-quality metagenomic assembly genomes (MAG) that were closely related to genomes sequences of strains isolated either from seeds (e.g., *Pseudomonas parafulva*; Midha et al., 2016), leaves (e.g., *Pseudomonas graminis*; Behrendt et al., 1999) or roots (e.g., *Pseudomonas rhizosphaerae*; Peix et al., 2003). These results suggest that some of the seed-associated bacteria are able to survive in different plant habitats and therefore, they can be vertical or horizontally transmitted from the seeds to the adult plant. Potentially, these microbes could also reach the next seed generation through the vascular system of the plant or via the pollen grains. Interestingly, one MAG associated to radish seeds is related to a new species of nectar-inhabiting bacteria (*Rosenbergiella nectarea)*, which could indicate transmission of this bacterial population through the floral pathways thanks to insect pollinators.

Overall, our study provides novel insights into the dynamics of seed-associated microbial assemblage during the early stages of the plant life cycle. We observed that emergence influences both the structure and function of these assemblages by selecting some taxa belonging to the *Gammaproteobacteria* and a set of key functions that could be related to the successful colonization and persistence of these micro-organisms in this habitat. These functions could be in part related to copiotrophy, which then suggest that selection during germination and emergence would favor species with fast-growing capabilities in response to the increase in nutrient availability. This pattern corresponds to a broader ecological lifestyle and sets the stage for a more functional targeted hypothesis testing in plant emergence. As copiotrophic taxa are also common residents of soil-associated microbial assemblages (Leff et al., 2015), uncovering how seed-borne microorganisms resist to invasion by these soil taxa represents an interesting area of research. According to our analysis of multifunctional diversity it seems that some seedlings-associated microbial assemblages are more prone to invasion than other. This has now to be test *in situ*. Our analyses represent one of the first attempts to empirically assess changes in the microbial community during plant emergence and moves us toward a more mechanistic understanding of these shifts. Altogether, the data obtained in this study could be useful for the design of complex seed inoculums possessing plant-beneficial properties and will contribute to a more holistic understanding of biological processes involved in the assembly of the plant microbiota.

## Author Contributions

GT-C, M-AJ and MBa designed the study and made substantial contributions to the analysis and interpretation of the results. SB prepared all the plant material for the experiments and performed the DNA extractions for sequencing. OB and CG carried out the shotgun sequencing from all the samples. MBr carried out bioinformatics analysis and participated actively in the interpretation of the results. MBa and GT-C wrote the manuscript with input from the other authors. All authors read and approved the final manuscript.

## Conflict of Interest Statement

The authors declare that the research was conducted in the absence of any commercial or financial relationships that could be construed as a potential conflict of interest.

## Footnotes

1 https://iris.angers.inra.fr/galaxypub-cfbp

2 https://github.com/martialbriand/IRHS_EmerSys/tree/master/Torres-Cortes_etal_2018
