# Supplementary material for: Functional Microbial Features Driving Community Assembly During Seed Germination and Emergence
Source: Front Plant Sci. 2018 Jun 29;9:902. doi: 10.3389/fpls.2018.00902 (PMC6034153; doi:10.3389/fpls.2018.00902)
Supplement: Supplementary file 2 [file Presentation_1.PDF]

Supplementary Material

Functional Microbial Features Driving Community Assembly During Seed Germination and Emergence.

Gloria Torres-Cortés[1]*, Sophie Bonneau[1], Olivier Bouchez[2], Clémence Genthon[2], Martial Briand[1], Marie-Agnès Jacques[1] and Matthieu Barret[1]

[1] IRHS, INRA, AGROCAMPUS-Ouest, Université d'Angers, Beaucouzé, France

[2] INRA, US 1426, Castanet-Tolosan, France

*** Correspondence:**
Dr. Gloria Torres-Cortés
Gloria.Torres-Cortes@inra.fr

Supplementary Figures

A)

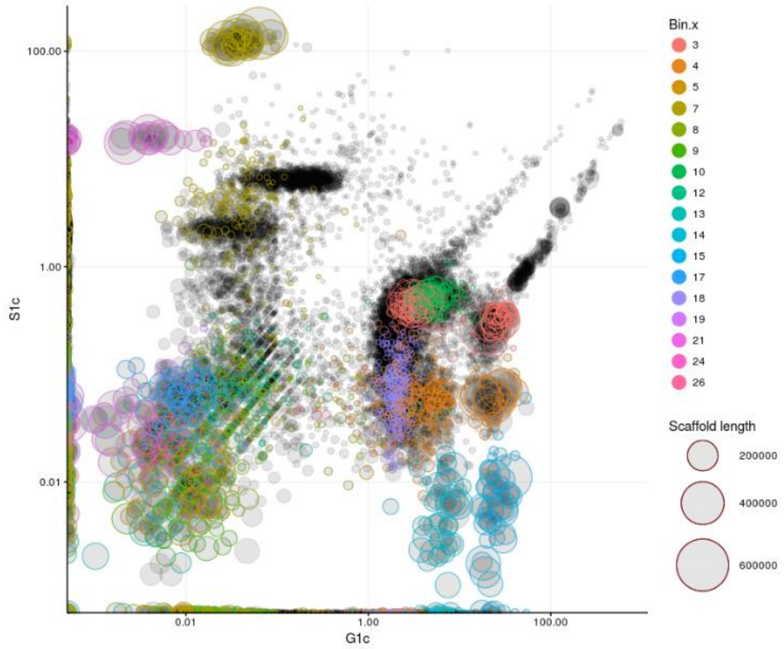

B)

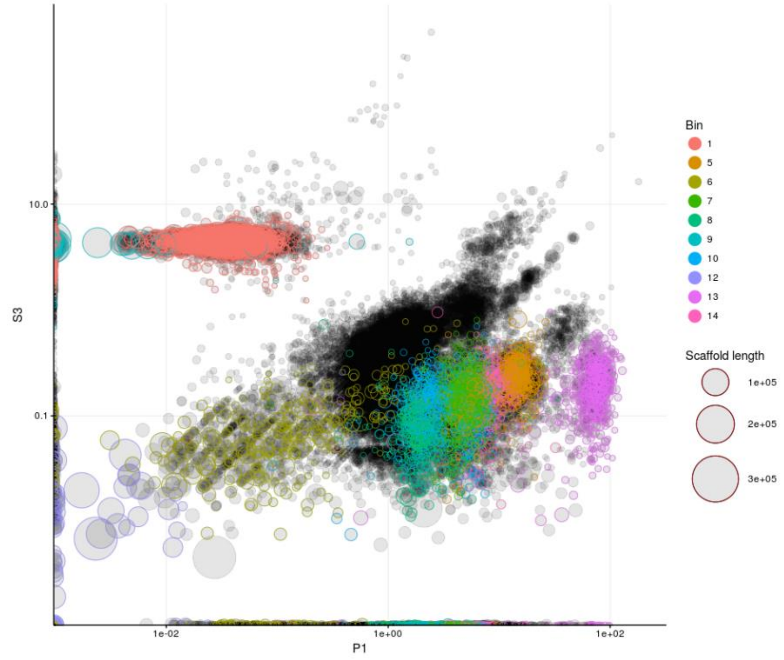

Supplementary Figure 1. Differential coverage plot of MAG-associated contigs. Circles represent contigs, scaled by the square root of their length and coloured by MAG groups. Only contigs >5kb are shown. (A) Bins reconstructed from bean metagenome datasets with differential coverage between germinating seeds (*x* axis; G1c) and seeds (*y* axis; S1c). (B) Bins reconstructed from radish metagenome datasets with differential coverage between seedlings (*x* axis; P1) and seeds (*y* axis; S3).

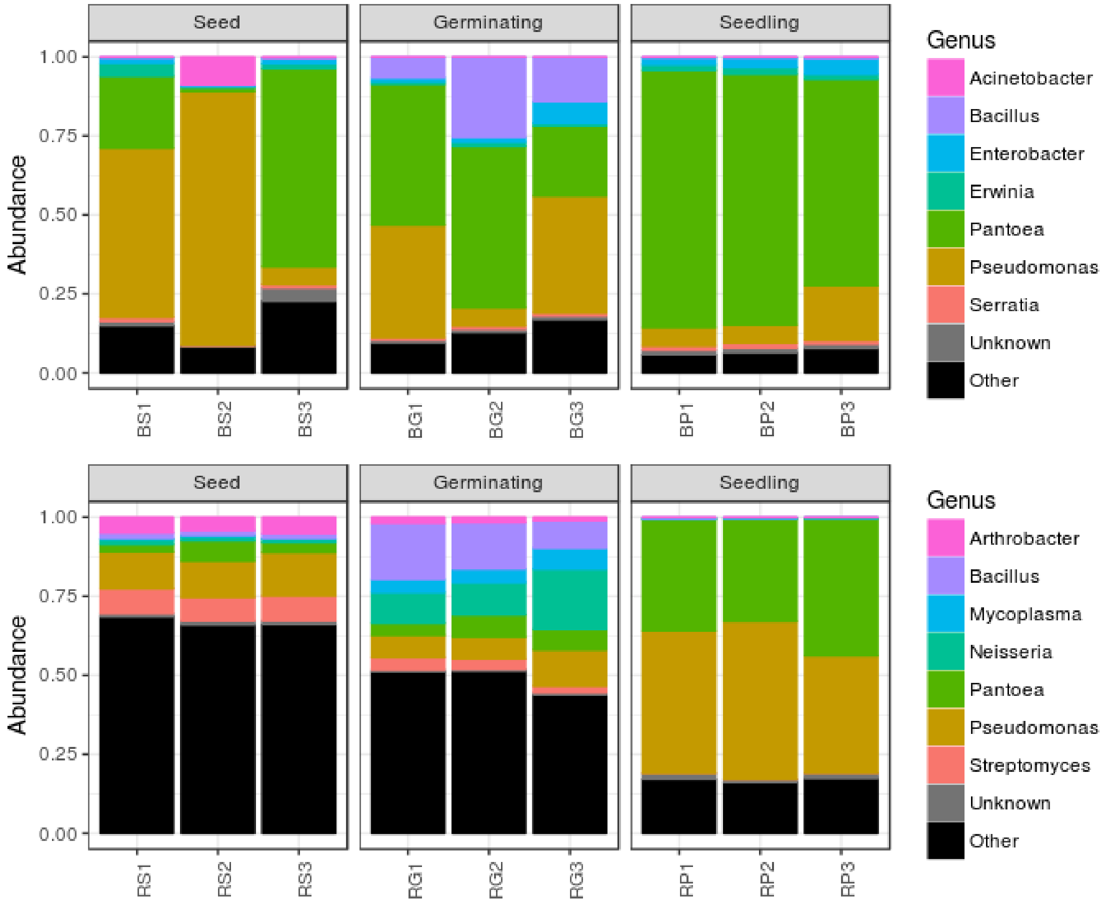

Supplementary Figure 2. Structure and composition of microbial assemblages associated to seeds, germinating seeds and seedlings. Phylogenetic assignment of reads in bacterial genus was performed with Clark, a k-mer based approach. Upper panel bean samples, lower panel radish samples.

A)

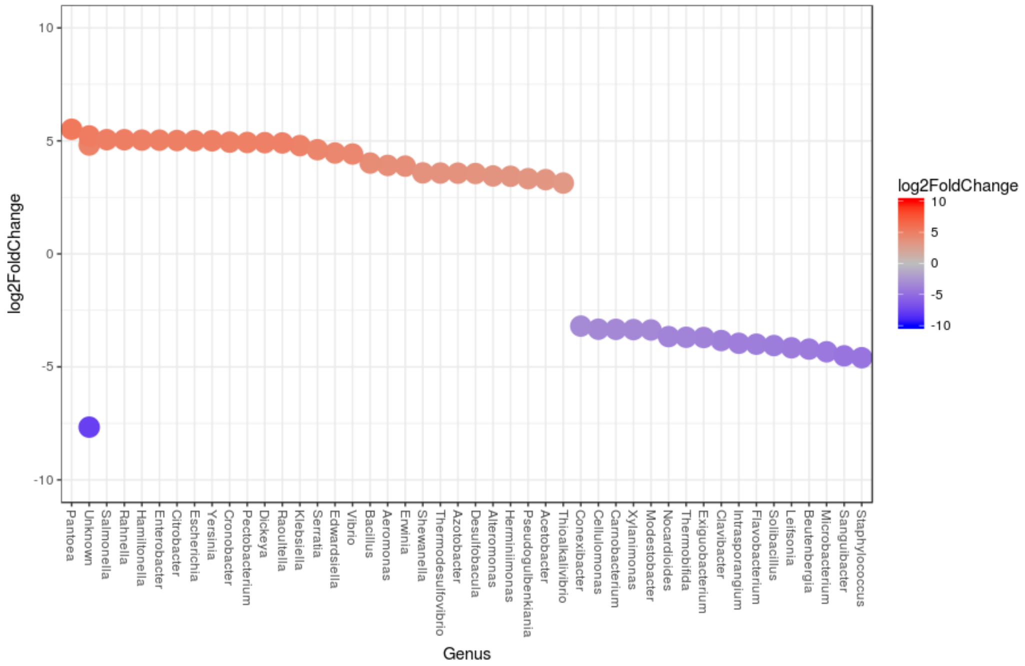

B)

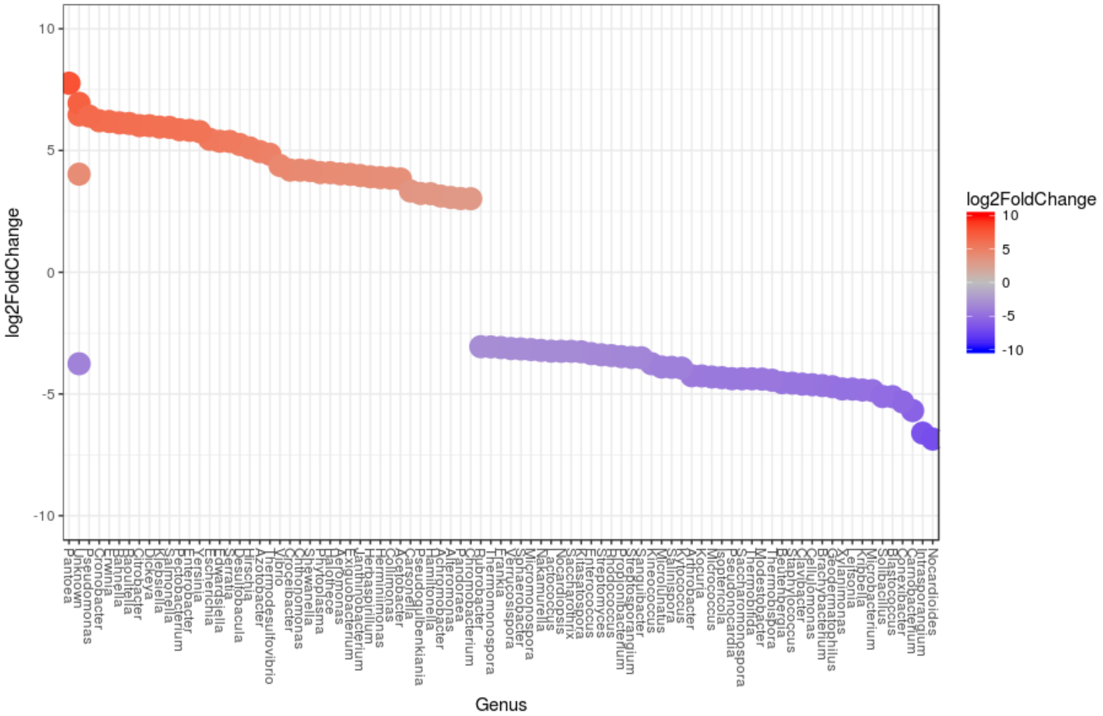

Supplementary Figure 3. Bacterial genera enriched and depleted in seedlings. Differences in relative abundance of each bacterial genus was assessed with DESeq2. Genera significantly enriched/depleted in seedlings in comparison to seeds ($P < 0.01$; logFC >3) are displayed for bean (A) and radish (B).

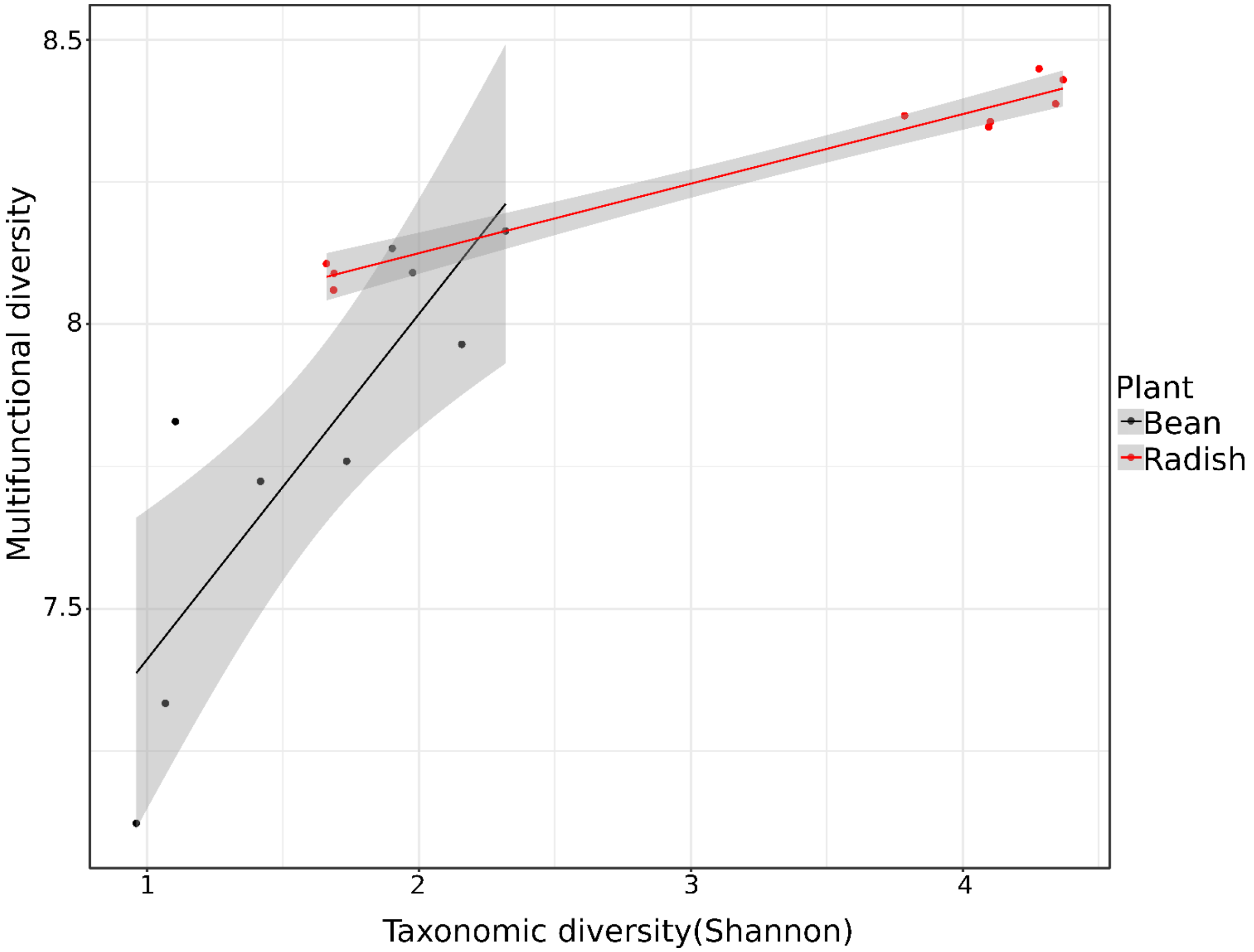

Supplementary Figure 4. Relationship between taxonomic and functional diversity. Accumulation curves showing the relationship between Shannon taxa diversity and Shannon multifunctional diversity with 95% of confidence interval

A)

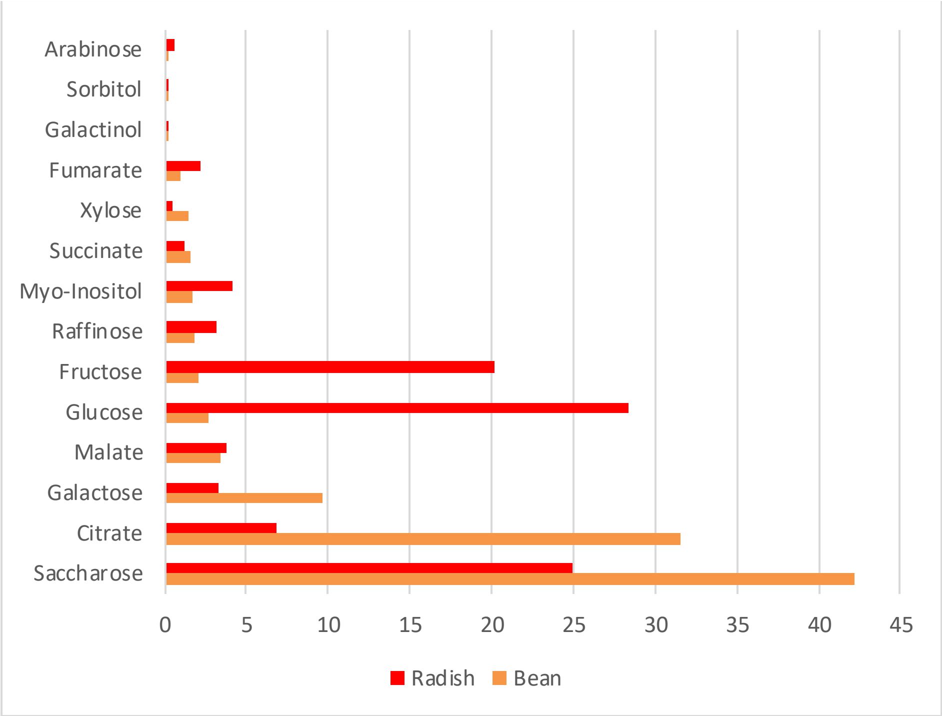

B)

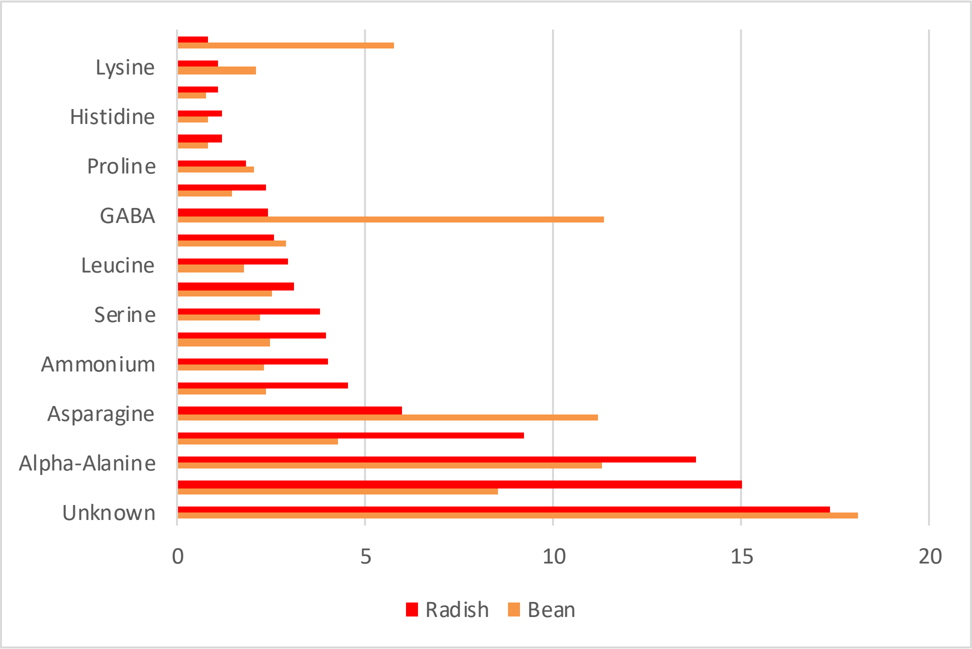

Supplementary Figure 5. Relative proportion of carbohydrate (A) and amino acid (B) in bean and radish seed exudates.

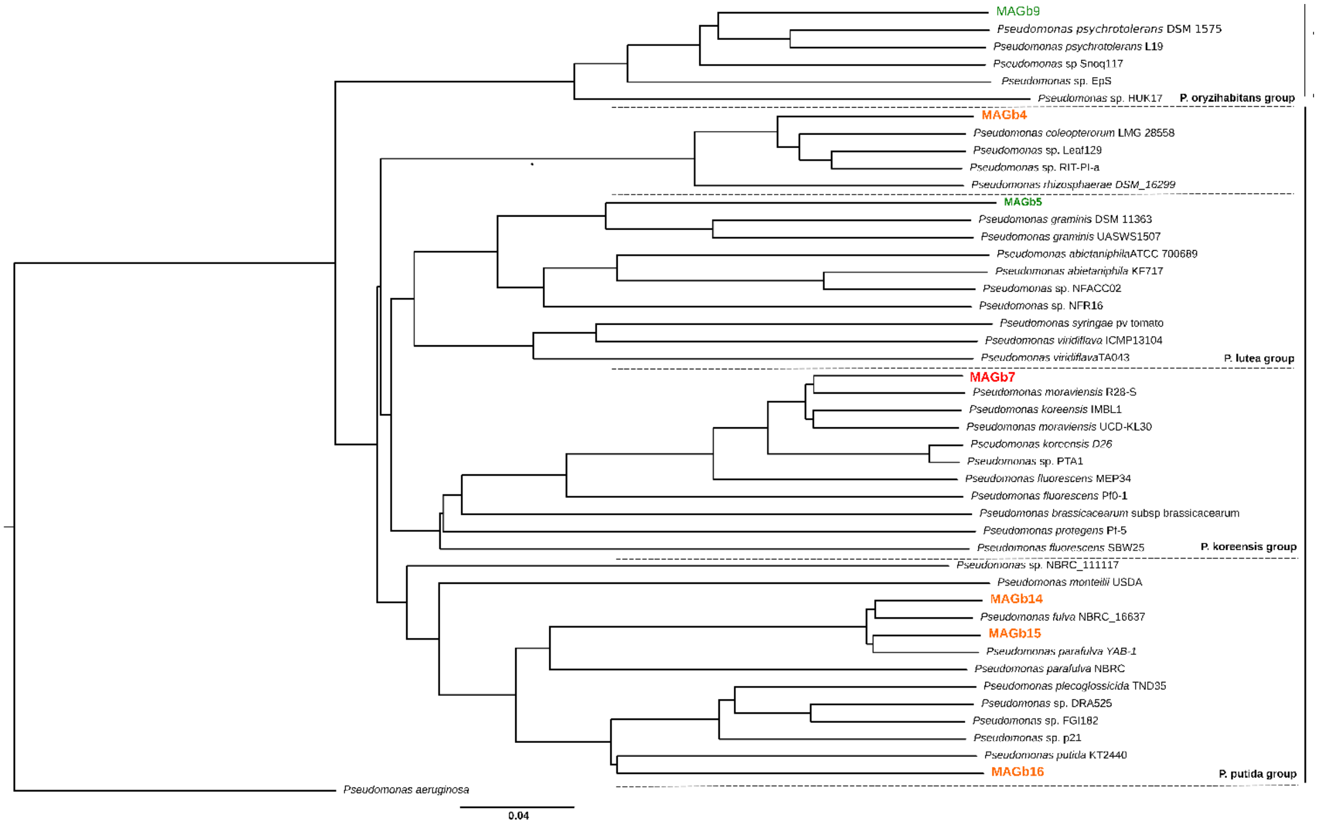

Supplementary Figure 6. Whole-genome based phylogenetic tree of *Pseudomonas*-affiliated MAGs. Composition Vector (CV) approach was performed on predicted proteomes of MAGs and relative genome sequences.

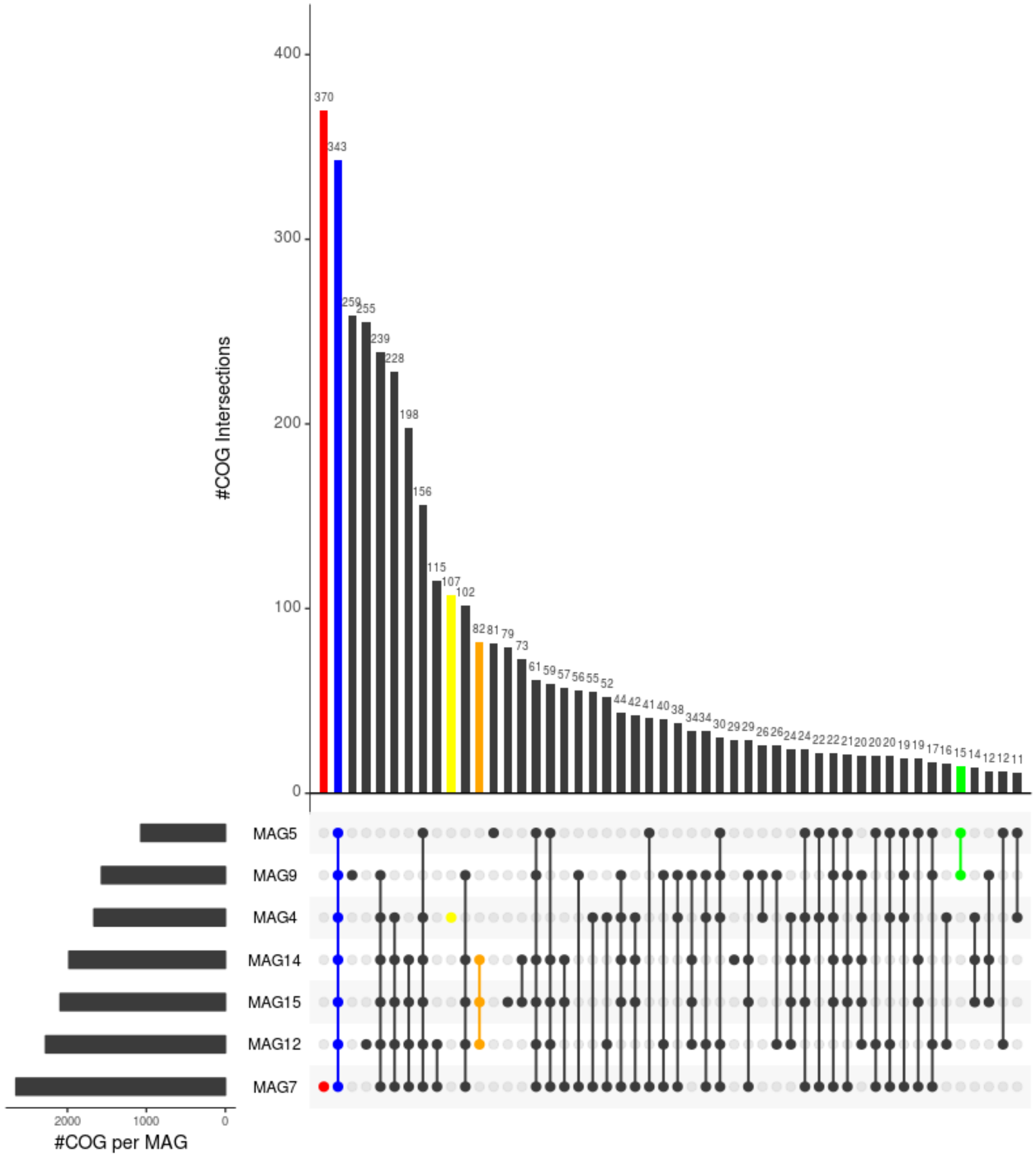

Supplementary figure 7. UpSet plot showing COG intersections between *Pseudomonas*-affiliated MAGs. The graphic presents the number of COGs shared between MAGs groups. Thus, the element view shows in blue the number of COG shared between all the MAGs, in orange the number of COG shared between germinating predominant MAGs (MAG14, MAG15 and MAG12) and in green the number of COG shared between the predominant MAGs in seedlings (MAG5 and MAG9).

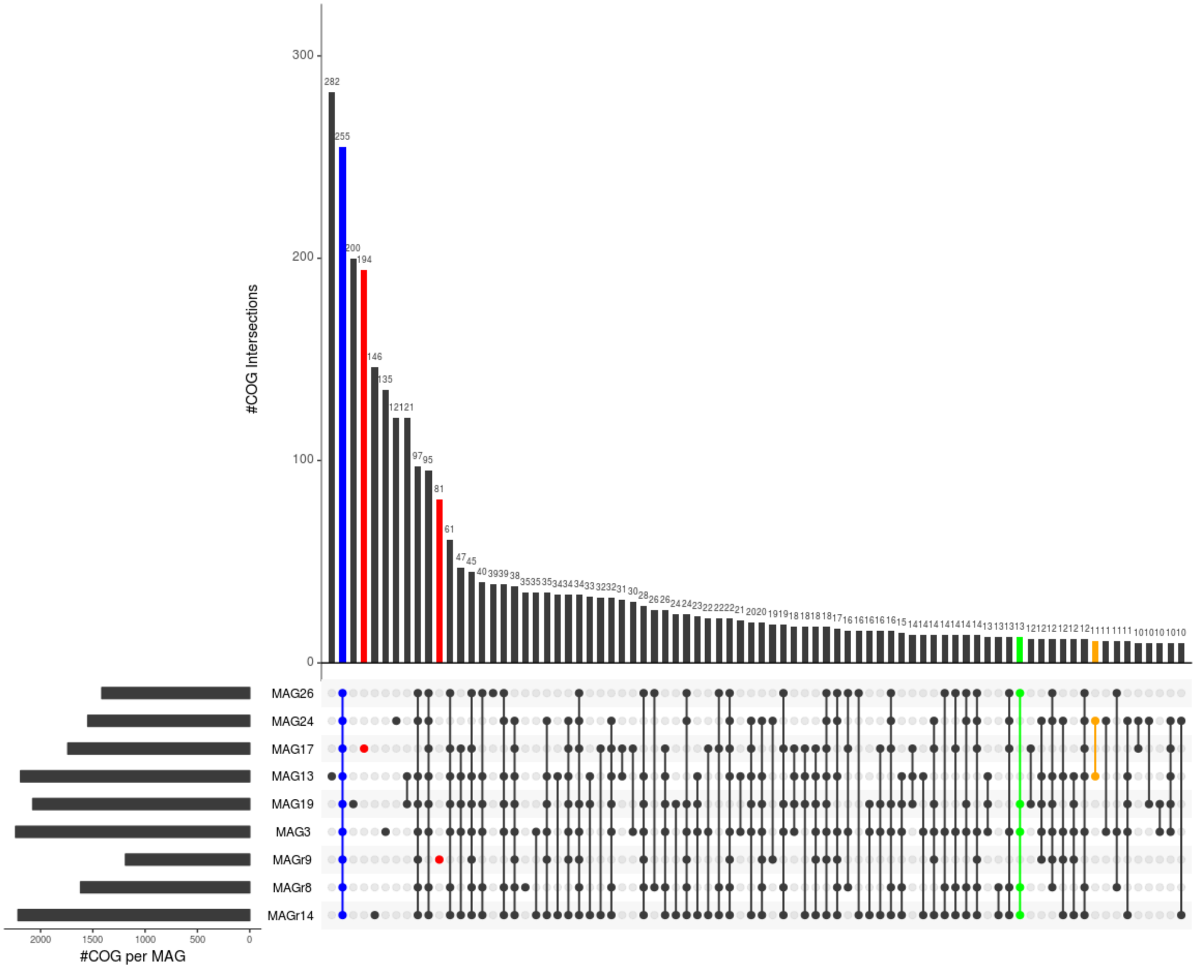

Supplementary figure 8. UpSet plot showing COG intersections between *Enterobacteriales*-affiliated MAGs. The graphic presents the number of COGs shared between MAGs groups. Thus, the element view shows in blue the number of COG shared between all the MAGs, in orange the number of COG shared between germinating predominant MAG (MAGb24 and MAGb13) and in green the number of COG shared between the MAG predominant in seedlings (MAGb26, MAGb19, MAGb3, MAGb8 and MAGr14).
